# A web-based intervention to promote applications for rehabilitation: a study protocol for a randomized controlled trial

**DOI:** 10.1186/s13063-015-0968-7

**Published:** 2015-09-29

**Authors:** Katja Spanier, Marco Streibelt, Firat Ünalan, Matthias Bethge

**Affiliations:** Institute of Social Medicine and Epidemiology, University of Lübeck, Ratzeburger Allee 160, 23562 Lübeck, Germany; Department of Rehabilitation, German Federal Pension Insurance, Ratzeburger Allee 160, 23562 Lübeck, Germany

**Keywords:** Rehabilitation, Need for rehabilitation, Web-based information, Health action process approach, Work ability index

## Abstract

**Background:**

The German welfare system follows the principle “rehabilitation rather than pension,” but more than the half of all disability pensioners did not utilize medical rehabilitation before their early retirement. A major barrier is the application procedure. Lack of information about the opportunity to utilize rehabilitation services restricts the chance to improve work ability and to prevent health-related early retirement by rehabilitation programs. The establishment of new access paths to medical rehabilitation services was, therefore, identified as a major challenge for rehabilitation research in a recent expertise. Thus, a web-based information guide was developed to support the application for a medical rehabilitation program.

**Methods/Design:**

For this study, the development of a web-based information guide was based on the health action process approach. Four modules were established. Three modules support forming an intention by strengthening risk perception (module 1), positive outcome expectancies (module 2) and self-efficacy (module 3). A fourth module aims at the realization of actual behavior by offering instructions on how to plan and to push the application process. The study on the effectiveness of the web-based information guide will be performed as a randomized controlled trial. Persons aged 40 to 59 years with prior sick leave benefits during the preceding year will be included. A sample of 16,000 persons will be randomly drawn from the registers of 3 pension insurance agencies. These persons will receive a questionnaire to determine baseline characteristics. Respondents of this first survey will be randomly allocated either to the intervention or the control group. Both study groups will then receive letters with general information about rehabilitation. The intervention group will additionally receive a link to the web-based information guide. After 1 year, a second survey will be conducted. Additionally, administrative data will be used to determine if participants apply for rehabilitation and finally start a rehabilitation program. The primary outcomes are the proportion of applied and utilized medical rehabilitation services. Secondary outcomes are cognitions on rehabilitation, self-rated work ability, health-related quality of life and perceived disability, as well as days with sick leave benefits and days of regular employment.

**Discussion:**

The randomized controlled trial will provide highest ranked evidence to clarify whether theory-driven web-based information supports access to rehabilitation services for people with prior sickness benefits.

**Trial registration:**

German Clinical Trials Register (Identifier: DRKS00005658, 16 January 2014).

**Electronic supplementary material:**

The online version of this article (doi:10.1186/s13063-015-0968-7) contains supplementary material, which is available to authorized users.

## Background

Many Western welfare systems provide rehabilitation services to improve work ability and to support participation in working life. In Germany, these services are provided usually as 3-week inpatient or outpatient programs under the umbrella of the German Pension Insurance (GPI). The treatment is multi-professional and follows evidence-based therapy standards [1]. The legal framework of the German rehabilitation system, which regulates access to and the organization of services, is based on the notion of preventing permanent work disability by the provision of rehabilitation services [2]. However, in 2013 58.6 % of all male and 51.9 % of all female first-time disability pensioners did not use rehabilitation services before their health-related early retirement [3]. In many cases the concept of “rehabilitation rather than pension” apparently does not work [4].

One main reason for non-utilization seems to be that medical rehabilitation requires a formal application of the person in need. A current study revealed that about one third of the disability pensioners were not informed on the possibility of claiming a medical rehabilitation program due to limitations in work ability [5]. If need for rehabilitation does not lead to an application of medical rehabilitation, the opportunity of preventing permanent work disability and the receipt of disability pensions remains unused. Though the Social Security Code explicitly states that guidance and motivation to use medical rehabilitation are obligatory if disabled people are in need (§26, Subsection 3, Sentence 7, SGB IX), there is obviously a lack of professional information on how to claim a medical rehabilitation [6]. The exploration and testing of new access paths to medical rehabilitation services were, therefore, identified as major challenges for rehabilitation research [7].

One potential gate for information might be web-based information guides [8]. These provide the advantage of easy access, even for persons having only scarce time resources or living in rural or in isolated areas. To use web-based information guides, only Internet access and a stationary or mobile Internet device is needed [9]. Recent studies point out the easy accessibility of the Internet among the working aged population and showed its relevance as a source for information search. In 2014, 94 % of Internet users aged 30 to 49 years and users aged ≥ 50 years utilized the Internet for seeking information [10]. Furthermore, web-based information services are less costly than compared with conventional information which makes use of paper, compact-discs or similar mediums [9].

Additionally, web-based information guides have the potential to support e-health literacy if they provide evidence-based information in a comprehensible format. This might help to handle the information flood of the Internet which often is perceived to be diffuse or overtaxing [11–13]. As a consequence, the relevance of the Internet as a medium of health-related information and interventions has substantially increased during recent years [9, 14–18].

In a qualitative study, Sillence et al. [13] examined factors supporting user acceptance and confidence in web-based information guides. Fussy layouts, confusing navigation, too much text and pop-ups increased rejection and mistrust towards a website. Web-based information guides were positively rated if they included interactive elements, e.g. assessments, clear and simple language, a section for frequently asked questions and unbiased information. Information provided by web-based guides, which were designed accordingly, was recognized as similarly important as information provided by relatives or physicians. However, general practitioners and specialists were still the first choice if a final decision was needed. These findings are in line with recent randomized controlled trials. Web-based information guides increased disease knowledge and health literacy and contributed to a reduction of uncertainty, anxiety and decision conflicts [19–22]. However, though web-based information seems to assist and prepare the decision-making process, it does not necessarily lead to a higher frequency of reached decisions either in favor of, or against, the intervention of interest than compared to a control group [19, 23].

Against this background we developed a web-based information guide promoting the application for rehabilitation services. The information guide aims at raising the frequency of applications for and utilization of medical rehabilitation services in persons who experience restrictions in their work ability. This study protocol describes the design of a randomized controlled trial which was developed to clarify whether the anticipated higher rates of applications and utilization will be achieved.

## Methods

### Design

The study will be performed as a randomized controlled trial.

## Treatment

### Control

Participants in the control group will receive a letter which informs about the general aims of medical rehabilitation and the possibility to apply for a medical rehabilitation program. The complete wording of the letter is available as Additional file 1.

### Intervention

Participants in the intervention group will also receive an informative letter. The first section of this letter equals the letter of the control group. In addition, the participants will be informed on a web-based information guide, obtain a link to the corresponding webpage and will be invited to use the web content in order to verify a potential need for rehabilitation and to claim a medical rehabilitation program. The complete wording of the letter is available as Additional file 2.

The development of the web-based information guide was based on the health action process approach (HAPA) [24]. According to the HAPA, health-related behavior change is described to be a process which consists of two phases: a motivational and a volitional phase. The motivational phase covers the growth of an intention to engage in a specific behavior. The volitional phase describes how an intention can be facilitated to turn into a specific behavior. During the motivational phase, the realization of an intention depends on the individual risk perception on what would happen without doing something, outcome expectancies in case of a specific behavior option and finally the level of perceived self-efficacy to act accordingly.

In the case of rehabilitation claims, Zimmermann et al. [25] reported that 41 % of persons with a self-rated need for rehabilitation intended an application for medical rehabilitation, and only 11 % of the persons with a subjective need finally applied for a rehabilitation program within 1 year. In a more recent study, Mittag et al. [26] reported comparable results. There seems to be both a lack of intention probably due to an information gap [6, 27] and a so-called intention-behavior gap. The first, the information gap, is partly reasoned by the segmented German health care system with different insurance schemes for primary health care and rehabilitation. Thus, important actors in the primary health care sector like the general practitioners have only limited knowledge and skills on the procedures which are needed to claim rehabilitation services [6]. The latter, the intention-behavior gap, refers to the well-known observation that an intention is not sufficient for realizing a specific behavior [28–30]. In this context, recent findings from health psychology research indicate that action planning is needed to translate health-related intentions into actual behavioral outcomes [24, 31, 32].

**Fig. 1:**
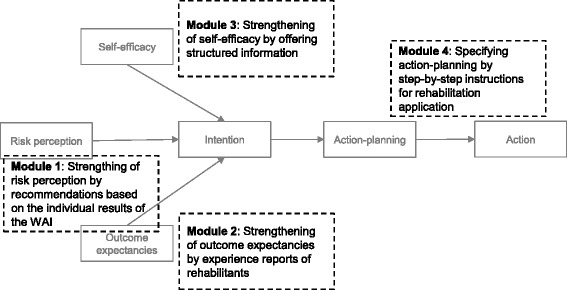
Conception of the web-based information guide

Thus, the web-based information guide was designed for supporting both, forming an intention and planning the application for medical rehabilitation. We therefore developed four modules. Modules 1 to 3 support the formation of an intention by strengthening risk perception (module 1), positive outcome expectancies (module 2) and self-efficacy (module 3). The realization of an actual behavioral outcome, e.g. a formal rehabilitation claim, is focused by the fourth module. This module includes structured instructions in order to support the planning of a rehabilitation claim (Fig. 1).

Staff members of the participating pension insurance agencies were consulted during the developmental process to include practical experiences and to consider the legal framework of the application process. Separate webpages were designed for each participating pension insurance agency because there are slight differences in some of the procedures and application forms. The web-based information guides are available at the web addresses http://www.reha-jetzt.de, http://www.reha2015.de and http://www.reha2016.de.

#### Module 1: completing the work ability index

Module 1 offers the option to evaluate the individual need for rehabilitation. To assess the potential need for rehabilitation the German version of the Work Ability Index (WAI) will be used.

The WAI was developed by the Finnish Institute of Occupational Health in the mid-1980s. It is available in 26 languages and internationally the most common instrument to assess work ability. The WAI measures the degree to which persons consider their state of health as adequate to cope with their job demands [33]. The WAI yields a score from 7 to 49, where higher scores indicate better work ability. These scores can be classified into 4 categories: poor (7–27), moderate (28–36), good (37–43) and excellent work ability (44–49) [34]. The authors of the WAI recommended tailored interventions for these categories, e.g. measures improving or restoring work ability if the current WAI is categorized as moderate or poor. According to the German social welfare system regulatory framework, both lower categories correspond with a need for rehabilitation [4].

Recent studies confirmed that the WAI is a predictor for the need for rehabilitation and rehabilitation-related impairments of functional capacity also within the setting of the German welfare system [4, 35]. These results are in line with international studies which identified the WAI as an important predictor of disability pension [36–38].

After completing the WAI the individual total score will be automatically calculated, and participants will receive a brief explanation and a concluding recommendation. The feedbacks are summarized in Table 1.

**Table 1:** Recommendations according to self-reported work ability

Work Ability Index: 37 to 49 points
Your work ability is good or excellent. At this moment, you do not have any health problems which significantly impair you in managing your daily work demands. You currently do not need a medical rehabilitation. But will it stay this way? The risk of coming down with chronic diseases increases with age. You should be prepared! Talk to your general practitioner or your occupational physician about opportunities how you can maintain your health and work ability.
Work Ability Index: 28 to 36 points
Your work ability is moderately limited. You suffer from health problems which make it difficult for you to cope with your daily work demands. Maybe you already stop enjoying work or worry about your future. A medical rehabilitation may support you in your current situation. Applying a rehabilitation is less laborious than what one might suppose. Our instructions will make it easy for you to master the application process. You will find the instructions right here.
Work Ability Index: 7 to 27 points
Your work ability is severely restricted. You suffer from health problems which make it impossible for you to cope with your daily work demands. Studies have shown that persons with low scores as yours have a remarkably high risk for premature retirement. But it is not too late to avoid this. You need a holistic treatment as provided by a medical rehabilitation program to manage your health problems. Applying a rehabilitation is less laborious than what one might suppose. Our instructions will make it easy for you to master the application process. You will find the instructions right here.

Persons with good or very good work ability currently do not have a need for rehabilitation. In this case the participants will receive recommendations how to maintain their work ability in cooperation with their general practitioner or their occupational physician. Participants with moderate work ability will be informed about medical rehabilitation as an option to improve the current work ability. If the WAI indicates poor work ability, participants will also receive a recommendation to apply for medical rehabilitation. Additionally, the participants are informed about the urgent need in order to avoid a disability pension. In both cases of recommendation for rehabilitation participants will be directly forwarded to the step-by-step instructions on how to apply for a medical rehabilitation.

#### Module 2: reports of positive experiences by rehabilitants

To strengthen positive outcome expectancies, reports of successful medical rehabilitation by former rehabilitation users will be presented. These reports shall strengthen the perception that rehabilitation is an effective option for the participants to sustain their work ability.

The reports are based on interviews which we conducted with former rehabilitants. Interview partners were recruited in five stationary rehabilitation centers and five ambulant rehabilitation centers. The guided interview dealt with the main diagnosis, personal pathways to rehabilitation, progress of health problems before, during and after medical rehabilitation and self-rated success factors of medical rehabilitation. Eleven guided interviews were conducted via phone and examined using content analysis [39]. For presentation on the webpage, contents were summarized as brief experience reports and enriched with original quotations from the interview. All experience reports are presented anonymized on the webpage. The experience reports were published only if the interview partners provided informed consent and agreed on using the interviews as examples at our web-based information guide. Participants of the intervention group may select and read each report individually.

#### Module 3: information on medical rehabilitation

In this module all relevant information on medical rehabilitation under the umbrella of the GPI and the application procedures are summarized in a clear and structured way. The content includes aims and types of rehabilitation as well as the legal framework and practical aspects, e.g. assumption of costs, continued payment of wages, additional payments and childcare facilities. In this way the participants will be prepared for the application process and be made aware of access barriers and information myths. The information section is hyperlinked to the step-by-step instructions and a frequently asked questions section (FAQ section). The FAQ section is designed dynamically. Participants may ask questions using a contact form which will be answered by mail. Questions and answers will successively complete the FAQ section. Additionally, a search function was implemented for accessibility of each section of the webpage by entering a search term.

#### Module 4: step-by-step towards the rehabilitation application

The step-by-step instructions shall reveal that the way towards an application for a rehabilitation program is manageable and that formal obstacles and the temporal expenditure can be mastered. The instruction traces the complete path of the application process until the start of the rehabilitation program and thus strengthens action planning by decomposing the complex task of a rehabilitation claim. Each required form is named and explained regardless of whether it has to be completed by the applicant herself or himself, by the attending physician, the health insurance or the employer. By this, participants will keep track of their documents and can control the application process (e.g. making an appointment with the attending physician for the medical statement which is needed). The description of the forms provides detailed explanations on how to complete them. Each administrative term and the function of the information that is to be given by the applicant will be explained. Additionally, a hyperlink is provided to download the corresponding form. All in all, the participants receive a comprehensive overview of the complete application process. This also includes the steps following the approval.

### Participants

Persons aged 40 to 59 years with prior sick leave benefits and regular employment of at least 6 months in the year before the beginning of the study will be included. Exclusion criteria are the receipt of pension benefits and the receipt of medical or vocational rehabilitation services during the last 4 years. Samples are randomly drawn from the registers of three pension insurance agencies, i.e. the federal agency and two regional agencies. Sampling will be stratified by pension insurance agency, sex and duration of sick leave benefits.

Persons will be informed about purpose and intervention of the study, its duration and data protection. Participation in the study will be consented by answering the questionnaire of the initial survey. Consent for using administrative data of the participants will be obtained by signature on the informed consent form during the second survey.

### Randomization

A postal survey on baseline characteristics will be sent to potential participants. Respondents to this first survey will be randomly allocated to the intervention and the control group. Randomization will be conducted by a statistician of the Institute of Social Medicine and Epidemiology, University of Lübeck, who is not otherwise involved with the trial, by using computer-generated random numbers. Allocation will be stratified by a pension insurance agency.

### Sample size estimation

To detect a 10-point higher rate of applications for a medical rehabilitation program (controls: 50 %), 1,076 persons (538 per group) are needed relying on a 2-sided alpha error of 0.05 and a power of 0.90. Response rates of 30 % to the first survey and of 50 % to the second survey were assumed to calculate the gross sample size to be drawn from the insurance registers. We finally decided to draw 8,000 persons for both the federal agency and the two regional agencies to allow segmented analyses according to the type of agency. Sample drawing was stratified by sex and the duration of sickness benefits in the year prior to the first survey (15 to 30 days versus more than 30 days).

### Data management

The insurance agencies generate numeric pseudonyms as unique identification numbers for all randomly selected individuals. The insurance agencies will post the pseudonymized questionnaires to the sampled individuals. Persons will be informed that in case of participating in the first survey they will be randomly allocated either to the intervention or the control condition. Participants will then send back their questionnaires to the researchers. After the first survey is completed, random assignment will be performed, and participants will obtain their corresponding intervention letter. Intervention letters for both the participants of the control group (see Additional file 1) and the intervention group (see Additional file 2) are available for more details. One year later, a second survey will be conducted. Moreover, the insurance agencies will provide administrative data on rehabilitation events to the researchers if participants give their approval. The numeric identification numbers will be used by the researchers to match questionnaire and administrative data. Consequently, researchers’ access to personal data of the sampled individuals (e.g. addresses and insurance account numbers) is avoided. Additionally, access and utilization of the webpage will be documented during the study period and provided as aggregated data by the open analytics platform Piwik (http://piwik.org).

## Outcome measures

### Main study outcome

The primary outcomes are the proportion of applied and utilized medical rehabilitation services after 1 year in the intervention group as compared to the control group. These data will be extracted from the individual GPI accounts.

### Secondary outcome

Secondary outcomes will be assessed by questionnaire after 1 year. These outcomes include cognitions on rehabilitation and the application process as well as indicators of work ability, health-related quality of life and perceived disability. Further secondary outcomes are days with sick leave benefits and days of regular employment during the 1-year follow-up. These data will be also extracted from the individual GPI accounts.

#### Attitudes toward medical rehabilitation and application of rehabilitation

Attitudes toward medical rehabilitation and the application process which will be assessed are related to positive and negative outcome expectancies, perceived self-efficacy to manage an application and self-rated level of information.

Positive outcome expectancies, e.g. managing work demands after the rehabilitation, will be assessed by four 5-point scaled items (1 = “absolutely agree” to 5 = “absolutely disagree”). In addition, work-related and family-related negative outcome expectancies, e.g. fear to be seen by co-workers as a slacker and uncertainty about who will keep obligations at home, will be assessed each by three items. Perceived self-efficacy to manage an application, e.g. certainty to apply for a rehabilitation even if it is complicated, will be assessed by four 4-point scaled items (1 = “agree” to 4 = “do not agree”) following Schwarzer et al. [24]. Finally, the current level of information about the application process, e.g. to be well-informed which documents are needed for the rehabilitation application, will be assessed by four 4-point scaled items (1 = “agree” to 4 = “do not agree”).

#### Self-rated work ability and disability days

Self-rated work ability will be assessed by the Work Ability Score (WAS). This is the first item of the WAI and compares current self-rated work ability to the lifetime’s best. The item has an 11-point scale, where 0 represents complete incapacity to work and 10 represents lifetime best work ability. The WAS correlates highly with the overall WAI score [33, 40]. Additionally, we will ask for the number of days during the last 6 months the participants have been kept from their usual work and housework activities (disability days) [41].

#### Health-related quality of life

Health-related quality of life will be assessed with 3 subscales of the 36-item Short-Form Health Survey of the Medical Outcomes Study (SF-36; physical functioning scale, mental health index and general health perception scale). The values of the scales range from 0 to 100. Higher values represent better health-related quality of life [42].

#### Sick leave benefits and regular employment

Information about days of sick leave benefits and days of regular employment will be extracted from the individual GPI accounts.

### Statistical analysis

The proportions of applied and utilized medical rehabilitation services will be compared by Pearson’s chi-squared test. Effects on continuous outcomes will be tested by analyses of covariance; thereby the baseline scores will be considered as a covariate [43].

### Ethical approval

The study protocol was approved by the ethics committee of the Hannover Medical School, Hannover, Germany (Number: 2060-2013). The committee stated that there are no legal or ethical concerns to the planned procedures. The trial was registered in the German Clinical Trials Register on 16 January 2014 (Identifier: DRKS00005658).

## Discussion

Our randomized controlled trial will provide highest ranked evidence to clarify whether theory-driven web-based information supports access to rehabilitation services for people with prior sickness benefits.

## Trial status

Conception and development of the web-based information guide is finished. Recruiting has started and is ongoing.

## Additional files

Additional file 1:
**Cover letter for participants of control group.** Translated version of the cover letter for participants of the control group. (DOCX 13 kb) Additional file 2:
**Cover letter for participants of the intervention group.** Translated version of the cover letter for participants of the intervention group. (DOCX 13 kb)

## Abbreviations

FAQ: frequently asked questions
GPI: German Pension Insurance
HAPA: health action process approach
SF-36: 36-Item Short-Form Health Survey
WAI: Work Ability Index
WAS: Work Ability Score
