# Supplementary material for: A web-based intervention to promote applications for rehabilitation: a study protocol for a randomized controlled trial
Source: Trials. 2015 Sep 29;16:436. doi: 10.1186/s13063-015-0968-7 (PMC4588912; doi:10.1186/s13063-015-0968-7)
Supplement: Additional file 2: — Cover letter for participants of the intervention group. Translated version of the cover letter for participants of the intervention group. (DOCX 13 kb) [file 13063_2015_968_MOESM2_ESM.docx]

**Additional file 2**

**Cover letter for participants of the intervention group**

Dear Sir or Madam,

About three months ago you received and answered a questionnaire from your Pension Insurance Agency. We would like to thank you for this and your readiness to participate in our study.

Maybe you fear being soon unable to work due to health problems or to pursue your profession until retirement. Maybe you are currently on sick-leave for a longer time. Many people do not know it, but a medical rehabilitation can help you in that case. Your Pension Insurance Agency provides these services to improve and to restore your work ability and to avoid a premature retirement.

A new webpage informs you about medical rehabilitation and the application procedure.

Together with the University of Lübeck we developed this webpage to assist you in your application. You can find out in a fast and easy test how strongly your work ability is impaired by your health problems.

In this case, you possibly are a candidate for a medical rehabilitation provided by your Pension Insurance Agency. The webpage www.reha2015.de (www.reha-jetzt.de, www.reha2016.de) leads you step-by-step through the application process until your application is ready for submission. Instructions will ease completing the forms. Additionally, all information you need about rehab is gathered on this webpage. Furthermore you get to know people who share their experiences with rehab with you and tell you how a medical rehabilitation had helped them.

If you apply for a medical rehabilitation we will consider your application in the usual manner and approve your rehab application if all conditions are met.

Yours sincerely
